# Palmitoyl Carnitine-Anchored Nanoliposomes for Neovasculature-Specific Delivery of Gemcitabine Elaidate to Treat Pancreatic Cancer

**DOI:** 10.3390/cancers15010182

**Published:** 2022-12-28

**Authors:** Akanksha Patel, Aishwarya Saraswat, Harsh Patel, Zhe-Sheng Chen, Ketan Patel

**Affiliations:** College of Pharmacy and Health Sciences, St. John’s University, Queens, NY 11439, USA

**Keywords:** Gemcitabine Elaidate, Protein kinase C, Palmitoyl-DL-carnitine chloride, pancreatic cancer

## Abstract

**Simple Summary:**

Despite the availability of numerous therapeutic approaches, pancreatic ductal adenocarcinoma still stands as one of the most fatal cancers worldwide. Nanoliposomes could potentially be helpful in improving the pharmacokinetic and anticancer efficacy of drug molecules. Gemcitabine is the first line of therapy, but due to its hydrophilic nature, entrapment within nanoliposomes is very limited. To overcome this problem, we explored the lipophilic form of gemcitabine, i.e., Gemcitabine Elaidate. The present study is aimed towards investigating the antitumor activity of Gemcitabine Elaidate and further designing an appropriate nano-liposomal formulation in combination with Palmitoyl-DL-carnitine chloride as a Protein kinase C (PKC) inhibitor. Our combination therapy in a nano-liposomal carrier resulted in enhanced cellular uptake, inhibition of angiogenesis potential and augmented anticancer potency in both 2D and 3D in vitro models of pancreatic tumors. The promising results indicate the successful development of a nano-liposomal carrier incorporating Gemcitabine Elaidate and Palmitoyl-DL-carnitine chloride as a more effective approach for the treatment of pancreatic cancer by overcoming the key drawbacks of the current first-line therapy.

**Abstract:**

Being the fourth most fatal malignancy worldwide, pancreatic cancer is on track to become the second leading cause of cancer-related deaths in the United States by 2030. Gemcitabine is a first-line chemotherapeutic agent for pancreatic ductal adenocarcinoma (PDAC). Gemcitabine Elaidate (Gem Elaidate) is a lipophilic derivative which allows hENT1-independent intracellular delivery of gemcitabine and better pharmacokinetics and entrapment in a nanocarrier. Cancer cells and neovasculature are negatively charged compared to healthy cells. Palmitoyl-DL-carnitine chloride (PC) is a Protein kinase C (PKC) inhibitor which also provides a cationic surface charge to nanoliposomes for targeting tumor neovasculature and augmented anticancer potency. The objectives of our study are: (a) to develop and characterize a PKC inhibitor-anchored Gem Elaidate-loaded PEGylated nanoliposome (PGPLs) and (b) to investigate the anticancer activity of Gem Elaidate and PGPLs in 2D and 3D models of pancreatic cancer. The optimized PGPLs resulted in a particle size of 80 ± 2.31 nm, a polydispersity index of 0.15 ± 0.05 and a ζ-potential of +31.6 ± 3.54 mV, with a 93.25% encapsulation efficiency of Gem Elaidate in PGPLs. Our results demonstrate higher cellular uptake, inhibition in migration, as well as angiogenesis potential and significant apoptosis induced by PGPLs in 3D multicellular tumor spheroids of pancreatic cancer cells. Hence, PGPLs could be an effective and novel nanoformulation for the neovasculature-specific delivery of Gemcitabine Elaidate to treat PDAC.

## 1. Introduction

Pancreatic ductal adenocarcinoma (PDAC) is the most invasive form of pancreatic cancer. It is currently the fourth most common cancer that results in death, and, by 2030, it is predicted to overtake lung and colon cancer as the second most lethal disease in the United States. In 2021, there were 48,220 deaths from pancreatic cancer, with 60,430 new cases reported by the American Cancer Society [1]. PDAC is not responsive to standard chemotherapy, surgery, targeted therapy, or immunotherapy; these treatments only provide modest-to-marginal additional improvements [2]. The current treatment strategies include albumin-bound paclitaxel (Abraxane), gemcitabine, 5-fluorouracil, irinotecan, single-agent therapy, and combinations of chemotherapy agents such as folfirinox [3,4].

The first-line treatment for pancreatic cancer is still gemcitabine (Gem), a DNA synthesis inhibitor. Even though therapeutic applications of Gem have demonstrated significant value, over time, its efficacy declines. This is mostly due to its short half-life, poor bioavailability, inadequate cellular absorption, and rapid degradation [5]. Due of its hydrophilicity, Gem needs transporter proteins to permeate across the cell membrane. There is proof that reduced cellular absorption caused by low nucleoside transporter proteins predicts the effectiveness of Gem [6]. Due to its strong affinity and efficiency to transport Gem, hENT1 is regarded as the major Gem carrier among the equilibrative nucleoside transporters [7]. In pancreatic ductal adenocarcinoma, the low expression of the human equilibrative nucleoside transporter-1 (hENT1) may lead to Gem resistance (PDAC) [8,9]. For overcoming this issue, the researcher developed Gemcitabine Elaidate (Gem Elaidate), which is chemically synthesized by covalently linking Gem and elaidic acid. The developed Gem Elaidate does not require hENT1 and can cross the biological membrane independently [9,10].

The Protein kinase C (PKC) family contains numerous isozymes with different roles in pancreatic cancer signaling pathways and commonly comprises various PKCs such as PKC-α, βI, βII and γ. Amongst these, PKCβ expression was observed to be substantially elevated in pancreatic ductal carcinoma [11]. The overexpression of PKC is directly correlated with the tumorigenicity of pancreatic cancer in vivo. PKC has been demonstrated to increase pancreatic cancer cell line proliferation and metastasis by downregulating PTEN and it has a role in the development of drug resistance in pancreatic cancer [12]. Most importantly, PKC triggers human endothelial cells’ angiogenic activity and produces VEGF, which in turn prompts it to release consistently through an autocrine positive feedback loop. Therefore, combining PKC inhibitor with an anticancer agent could be a new therapeutic strategy to deal with pancreatic tumors [13,14]. For improved cellular absorption and increased anticancer activity in PDAC, we employed Palmitoyl-DL-carnitine chloride (PC) as a Protein kinase C (PKC) inhibitor in the current investigation [15].

A reimagining is required for effectively delivering Gem Elaidate, which can be fulfilled with the help of the nanomedicine platform that helps to overcome the issues related to conventional drug formulations. Gem Elaidate, a lipophilic derivative, has very low water solubility. Therefore, formulating an oral formulation is problematic [16]. Due to their inadequate tumor site targeting, traditional chemotherapeutics were always associated with serious side effects in cancer treatment. PEGylated stealth liposomes are frequently employed as a nanocarrier to deliver anticancer medications because they evade the immune system and reticuloendothelial system (RES) detection and extend blood circulation [17,18,19]. Furthermore, nanoliposomes tend to aggregate in tumor tissue because of EPR-mediated passive tumor targeting, thus improving the efficacy of drug delivery [20]. Considering the above, liposomes are the appropriate choice to co-deliver Gem Elaidate and PC. The tumor-specific delivery of anticancer drugs is greatly influenced by the surface properties of liposomes. Cationic liposomes have been shown in prior research to accumulate more in tumor vasculature, improving the intratumoral delivery of chemotherapeutic agents [17,18]. Additionally, lactate production from anaerobic glycolysis is the cause of the acidic pH of solid tumors and an anionic charge is due to the presence of flipped phosphatidylserine and interstitial hyaluronic acid. Therefore, the cationic charge provided by PC to nanoliposomes and the anionic charge of vascular endothelial cells interact electrostatically, thereby improving cell penetration [21]. The purpose of this research is to develop PKC inhibitor-anchored Gem Elaidate-loaded PEGylated nanoliposomes (PGPLs) and to investigate their anticancer activity in 2D and 3D models of pancreatic cancer.

## 2. Materials and Methods

### 2.1. Materials 

Gemcitabine was procured from LC laboratories (Woburn, MA, USA), Gem Elaidate from Musechem (Fairfield, NJ, USA), Palmitoyl-DL-carnitine chloride from Chemcruz (Dallas, TX, USA), and 1,2-Dioleoyl-sn-glycero-3 phosphocholine (DOPC) and DSPE 18:0/18:0-PEG2000 from Lipoid (Ludwigshafen, Germany). Cholesterol and chloroform were purchased from Sigma-Aldrich (St. Louis, MO, USA). Other chemicals and materials are described in Supplementary Materials.

### 2.2. Cell Lines and Culture Conditions

MIA PaCa-2 and BxPC-3 human pancreatic cancer cells were obtained from the American Type Culture Collection, ATCC (Manassas, VA, USA). The MIA PaCa-2 cell lines were grown in DMEM media and BxPC-3 cells were maintained in RPMI 1640 with high glucose- L-glutamine–HEPES. Both media were supplemented with 10% FBS, 2 mM L-glutamine and 1 Mm sodium pyruvate, 1% penicillin-streptomycin and incubated at 37 °C and 5% CO2 with 95% relative humidity.

### 2.3. HPLC Analysis

The analytical method using HPLC for Gem Elaidate and Gem detection are described in detail in the Supplementary Materials.

### 2.4. Preparation of PGPLs

The PGPLs were prepared by the previously established modified hydration method and the detailed procedure is described in the Supplementary Materials [22,23,24].

### 2.5. Characterization and Stability Study of PGPLs

The particle size, polydispersity index (PDI), and zeta potential were measured using dynamic light scattering (DLS) (Malvern, Zetasizer Nano ZS, UK). For particle size measurements, disposable cuvettes, and for zeta potential, disposable folded capillary cell were used at 25 °C. To estimate the entrapment efficiency of the PGPLs, Amicon Ultra centrifugal filters (50 K) were used. The concentration of total and free drug was analyzed using HPLC. For calculating encapsulation efficiency, the following formula was used:

$$\begin{array}{c} Percent\;Encapulation\;efficiency=\frac{\left[Total\;drug\right]-\left[Free\;drug\right]}{\left[Total\;drug\right]}\times 100\\ Percent\;drug\;loading=\frac{\left[Amount\;of\;entrapped\;drug\right]}{\left[Total\;solid\;content\right]}\times 100 \end{array}$$

PGPLs prepared by the modified hydration method were subjected to a stability study at 4 °C. Particle size, PDI, and zeta potential were evaluated every week.

### 2.6. In Vitro Cytotoxicity

In vitro cytotoxicity of Gem Elaidate and Gem was evaluated in two pancreatic cancer cell lines (MIA PaCa-2 and BxPC3) using 3-(4,5-dimethylthiazol-2-yl)-2,5-diphenyl tetrazolium bromide (MTT) assay. The detailed procedure is described in the Supplementary Materials.

### 2.7. Quantitative Cellular Uptake Assay

Quantitative cellular uptake of Gem Elaidate, Gem, and PGPLs was determined in MIA PaCa-2 cells. the detailed procedure is described in the Supplementary Materials [25].

### 2.8. In Vitro Hemolysis Study

The in vitro hemolysis study of fabricated PGPLs at different concentration was carried out using mouse red blood cells (RBCs). The detailed procedure is described in the Supplementary Materials [26].

### 2.9. In Vitro Migration Assay

To determine the percent of the bridging of the migration area for Gem Elaidate, Gem and PGPL-treated cells, an in vitro migration assay was performed. the detailed procedure is described in the Supplementary Materials [27].

### 2.10. Clonogenic Assay

The effectiveness of Gem Elaidate, Gem, and PGPLs on colony inhibition for MIA PaCa-2 was examined using a clonogenic assay [28].

### 2.11. In Vitro Vasculogenic Mimicry Assay

For this assay, MIA PaCa-2 cell suspension with cell density of 1 × 10^4^ cells/well was incubated with Gem Elaidate (1 μM), PC (1 μM) and PGPLs (1 μM) on a precoated matrigel (50 µL) matrix plate. Using an EVOS light microscope at 10×, the formation of tubules in each field was captured and an average number of tubules was counted for 3–6 randomly chosen fields in each well to assess vasculogenic mimicry [15,29,30]

### 2.12. Western Blot Assay

Protein expression levels of p53, Bcl-2, cleaved caspase-3 in MIA PaCa-2 cells following treatment with Gem Elaidate and PGPLs were analyzed according to a previously established protocol for western blot [27,31]. The detailed methods are described in the Supplementary Materials.

### 2.13. Formation and Treatment of 3D Multicellular Tumor Spheroids

3D multicellular tumor spheroids were developed using a previously established protocol [31]. MIA PaCa-2 cells were seeded at a density of 1500 cells/well in an ultra-low attachment 96-well plate. Spheroid microplates were centrifuged at 130× *g* for 10 min and incubated for 3 days prior to treatment. The spheroids were treated with multiple doses of Gem Elaidate (2 μM), Gem (2 μM), and PGPLs (2 μM) with media as a control every alternate day up to 10 days. Images were captured before treatment each time for continuous observation of spheroids in terms of their diameter and surface area using an EVOS imaging system (Thermo Fisher Scientific, Waltham, MA, USA) at 10× magnification.

### 2.14. Cell Viability within 3D Multicellular Tumor Spheroids

The cell viability and 3D cell imaging were taken on day 10 of treatment. The detailed procedure is described in the Supplementary Materials.

### 2.15. Statistical Analysis

Each experiment was performed in triplicate, and all data are presented as the mean ± standard deviation (SD). Student’s *t*-tests or one-way ANOVA–Bonferroni tests were used to compare results using GraphPad Prism 7. (GraphPad Software, San Diego, CA, USA). A *p*-value of 0.05 indicated a statistically significant difference between the treatment groups.

## 3. Results

### 3.1. HPLC Analysis 

The developed HPLC method eluted Gem Elaidate and Gem within a 10 min run time, giving a sharp peak for the drug at 275 nm with a retention time of ~4.8 ± 0.2 min and 5.6 ± 0.1 min, respectively. The same HPLC method was used to analyze all the samples for entrapment efficiency and quantitative cellular uptake studies.

### 3.2. Characterization and Stability of PGPLs

**Figure 1:**
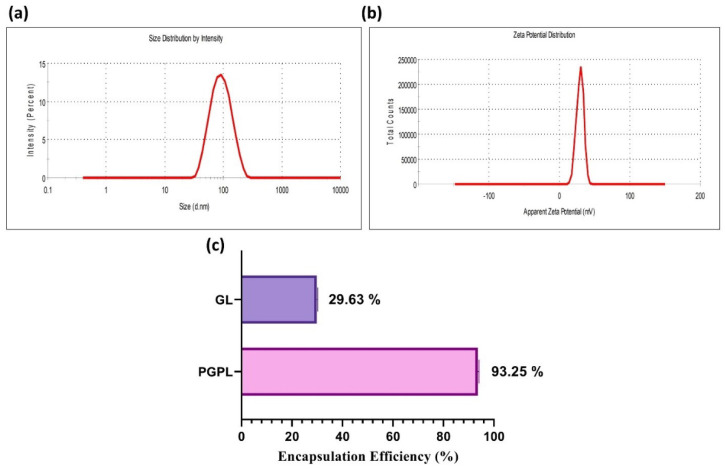
Physicochemical characterization of developed liposomes: (**a**) Dynamic light scattering graphs illustrating unimodal particle size distribution and (**b**) Positive ζ-potential of optimized PGPLs. (**c**) Entrapment efficiency comparison of Gem Elaidate and Gem in PGPLs and GL, respectively.

Particle size and zeta potential are vital aspects of drug delivery systems that may affect the permeability, uptake, and stability of nanoparticles. As shown in Figure 1a, the hydrodynamic diameter of the PGPLs was 81.78 ± 2.31 nm with a PDI less than 0.20. The zeta potential was found to be +31.6 ± 3.54 mV, which is attributed to carnitine on the surface (Figure 1b). At the same time, the particle size of the PGPLs without PC was more than 130 nm with a PDI of over 0.30 and a zeta potential of −46.4 ± 12.8 (Figure S1). The PGPLs were found to be stable in terms of particle size, polydispersity index, and zeta potential for two months at 4 °C. (Table 1) Moreover, an encapsulation efficiency of Gem Elaidate and Gem was found to be 93.25% *w*/*v* and 29.63% *w*/*v*. (Figure 1c). A 4.77% *w*/*w* drug loading of the PGPLs was achieved. Due to the surface active property of PC (it acts like a cationic surfactant due to carnitine), liposomes with PC were smaller in size.

### 3.3. In Vitro Cytotoxicity Test

The in vitro cytotoxicity of Gem Elaidate, Gem and PGPLs was evaluated in MIA PaCa-2 and BxPC3 cell lines. As shown in Table 2, the IC50 of the individual drug and formulation are similar in both cell lines. Gem Elaidate exhibited a ~3-fold lower IC50 compared to Gem. Gem Elaidate and PGPLs showed a comparable cytotoxicity nearly equal IC50 (Figure S2).

### 3.4. Quantitative Cellular Uptake Assay

**Figure 2:**
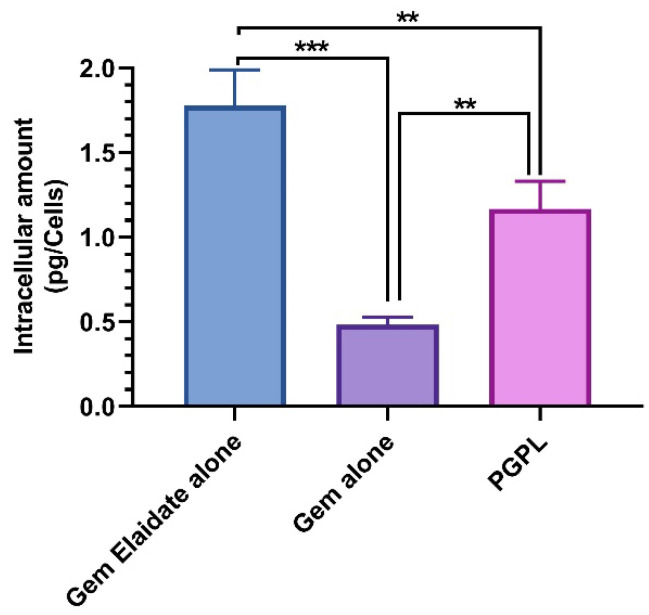
Quantitative analysis of intracellular amount of Gem Elaidate alone, Gem alone, and Gem Elaidate in PGPLs using MIA PaCa-2 cells incubated for 4 h at 37 °C. (*n* = 3) ** *p* < 0.01; *** *p* < 0.001.

Quantitative cellular uptake assay was performed to confirm the intracellular uptake of Gem Elaidate, Gem, and PGPLs in MIA PaCa-2 cells. As observed in Figure 2, the intracellular amount of Gem Elaidate was significantly higher in cells treated with Gem Elaidate alone (1.73 pg/cells) compared to PGPL (1.2 pg/cells). Moreover, the cellular uptake of the Gem was relatively low compared to both Gem Elaidate and PGPLs, which could be due to the hENT1-dependent intracellular delivery of Gem. Furthermore, similar results were obtained for intracellular uptake in BxPC-3 cells (Figure S3).

### 3.5. In Vitro Hemolysis Study

**Figure 3:**
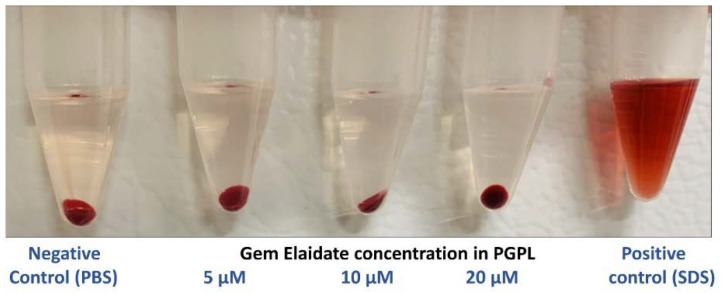
In vitro hemolysis study of PGPLs. PGPLs showed <1% cell lysis at different concentrations compared to positive control (SDS) (*n* = 3). PBS: Phosphate-buffered saline; SDS: Sodium dodecyl sulfate.

Through in vitro hemolysis assay, the effect of PGPLs on red blood cells was assessed. As shown in Figure 3, PGPLs showed negligible hemolysis (<1%) at different concentrations of Gem Elaidate (5 µM, 10 µM, and 20 µM) in contrast to the positive control (sodium dodecyl sulfate), which indicates 100% hemolysis. The fact that RBCs redispersed quickly and completely reveals that the PGPLs had not changed their surface properties.

### 3.6. In Vitro Migration Assay

**Figure 4:**
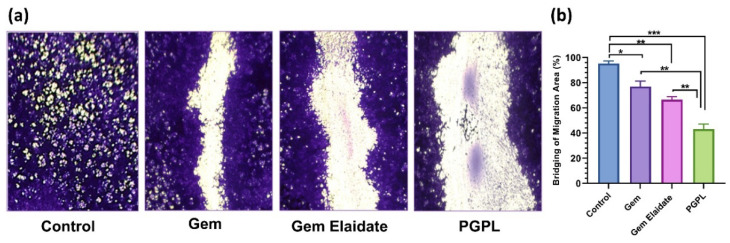
In vitro migration assay in MIA PaCa-2 cells. (**a**) Images of in vitro migration assay for Gem Elaidate, Gem, and PGPL-treated cells compared to control and captured at 10× (**b**) Percentage inhibition of migration produced by Gem Elaidate, Gem, and PGPLs in MIA PaCa-2 cells (*n* = 3). * *p* < 0.1; ** *p* < 0.01; *** *p* < 0.001.

The in vitro migration assay was used to investigate the molecular mechanism of cell migration. Figure 4a illustrates the scratch area of MIA PaCa-2 cells treated with 100 nM of Gem Elaidate, Gem, and PGPLs compared to the control group, respectively. The control cells completely bridged the scratch within 24 hrs. On the other hand, PGPLs and Gem Elaidate showed a significant percentage inhibition of cell migration, as shown in Figure 4b. Moreover, the results depict a significant inhibition of the scratch area on treatment with PGPLs, which was found to be 1.48-fold higher than that of Gem Elaidate alone.

### 3.7. Clonogenic Assay

**Figure 5:**
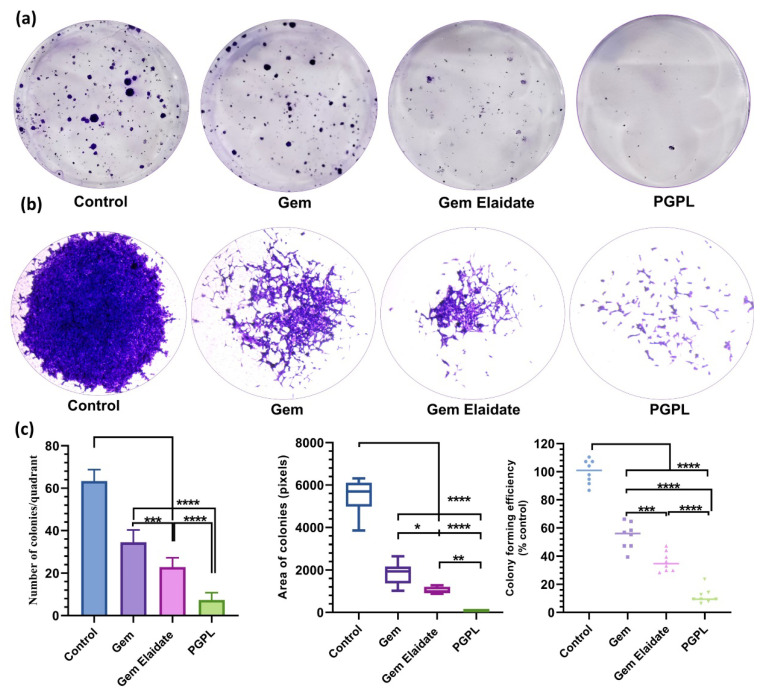
Clonogenic assay: (**a**) Crystal violet-stained images for Gem, Gem Elaidate, and PGPL-treated cells for a number of colonies. (**b**) Crystal violet-stained images for Gem, Gem Elaidate, and PGPL-treated cells for area of colonies formed. (**c**) Quantitative illustration of reduction in the number, area, and percentage decrease in colony forming efficiency with Gem, Gem Elaidate, and PGPL treatment in comparison to control. (*n* = 3). * *p* < 0.1; ** *p* < 0.01; *** *p* < 0.001; **** *p* < 0.0001 (comparison to control).

Cell survival after drug exposure was assessed by clonogenic assay. As clearly depicted in Figure 5a, in comparison to the control group, MIA PaCa-2 cells treated with Gem Elaidate, Gem, and PGPLs at a 50 nM concentration showed fewer colony formations. Additionally, PGPL-treated cells showed 3.5-fold and 4.6-fold reductions in the number of colonies formed when compared to Gem Elaidate alone and Gem, respectively (Figure 5c). If the colony area was taken into consideration, the cells treated with PGPLs show a 13-fold and 23-fold smaller colony area compared to the Gem Elaidate and Gem-treated cells respectively. Therefore, we can say that the area of colonies was drastically reduced by exposure to PGPLs compared to the control, Gem Elaidate, and Gem, as shown in Figure 5b. The colony forming efficiency of PGPLs is significantly lower compared to the control, as observed in Figure 5c.

### 3.8. In Vitro Vasculogenic Mimicry Assay

**Figure 6:**
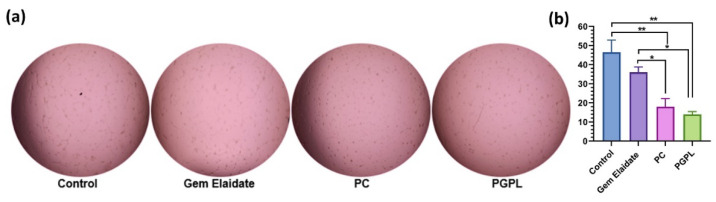
Evaluating the effect of PGPLs on MIA PaCa-2 cells’ vasculogenic mimicry. (**a**) Vasculogenic mimicry images of cells treated with Gem Elaidate (1 μM), PC (1 μM) and PGPLs (1 μM). (**b**) Number of branching points after treatment with Gem Elaidate, PC and PGPLs. (*n* = 3). * *p* < 0.1; ** *p* < 0.01.

Rapid tumor progression, development of drug resistance, and metastasis are promoted by the formation of vasculogenic mimicry [32]. Vasculogenic mimicry structures were observed in the control group for MIA PaCa-2 cells embedded on the ECM, whereas PC and PGPLs showed a drastic inhibition of VM channel formation (Figure 6a). Treatment with PC and Gem Elaidate significantly decreased the branching points, while the combination of Gem Elaidate with PC in PGPLs further disrupted the formation of vasculogenic mimicry (Figure 6b).

### 3.9. Western Blot Assay

**Figure 7:**
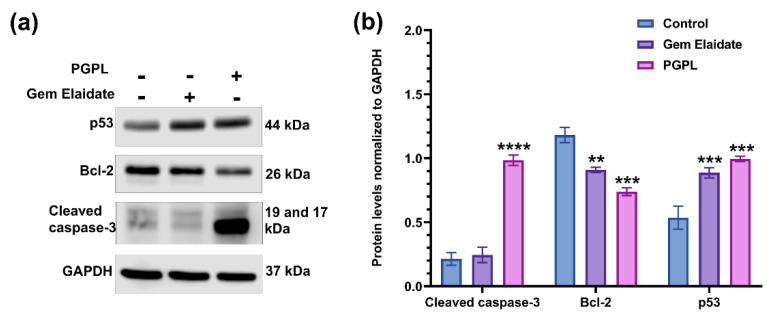
Western blot analysis results. (**a**) Expression of different proteins as determined by west-ern blot following Gem Elaidate and PGPL (200 nM) treatment in MIA PaCa-2 cells. (**b**) Quantitation of each protein expression. Protein levels were normalized to GAPDH (*n* = 3). ** *p* < 0.01; *** *p* < 0.001; **** *p* < 0.0001 (comparison to control).

As illustrated in Figure 7, the level of anti-apoptotic Bcl-2 protein expression decreased significantly in the presence of Gem Elaidate and PGPLs in MIA PaCa-2 cells in comparison to the control. Bcl-2 protein expression level was found to be substantially lower in PGPL-treated cells when compared to the Gem Elaidate treatment. Consequently, the expression levels of apoptotic marker-cleaved caspase-3 were found to be significantly higher following PGPL treatment, indicating the strong apoptotic effect of the Gem Elaidate-loaded nano-formulation in MIA PaCa-2 cells. Furthermore, the expression levels of the tumor suppressor protein p53 were found to be substantially higher in both Gem Elaidate and PGPL-treated cells compared to the control, implying their anticancer efficacy in PDAC (Figure S4).

### 3.10. Formation and Treatment of 3D Multicellular Tumor Spheroids

**Figure 8:**
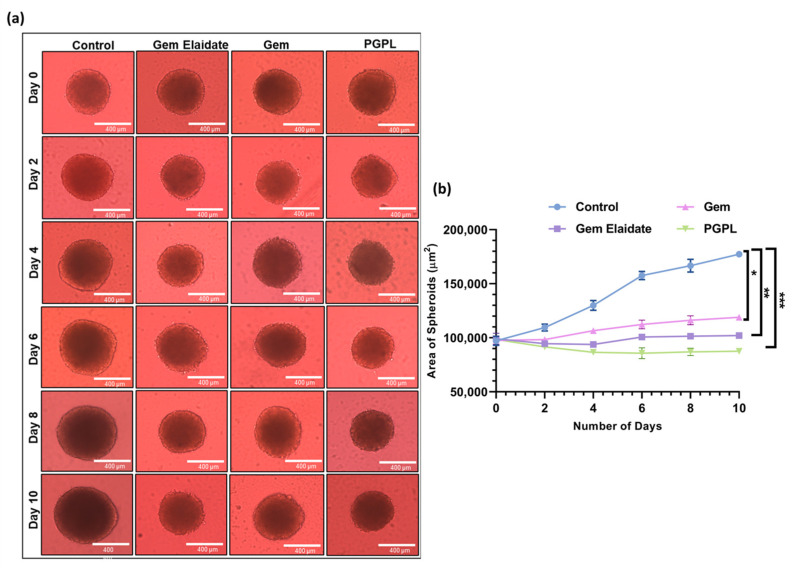
Results for cell viability within 3D multicellular pancreatic tumor spheroids of MIAPa-Ca-2. (**a**) Representative images of spheroids treated with Gem Elaidate, Gem, and PGPLs following 10 days of treatment. (**b**) Area of spheroids as a function of time with various treatment groups. (*n* = 3). * *p* < 0.1; ** *p* < 0.01; *** *p* < 0.001.

According to Figure 8a,b, the Gem Elaidate-treated group significantly inhibits cell proliferation, with a significant area reduction until day 4 and then a merely stagnant spheroid area until day 10 of treatment. Additionally, the PGPL treatment group’s spheroid surface area was significantly lower than that of the other groups, indicating that it plays a more significant role in cell death and growth suppression than Gem Elaidate alone or Gem. In contrast to the solid surface seen in the control group, the outer surface of spheroids for Gem Elaidate and PGPLs was highly uneven because of apoptotic cells on the surface.

### 3.11. Cell Viability within 3D Multicellular Tumor Spheroids

**Figure 9:**
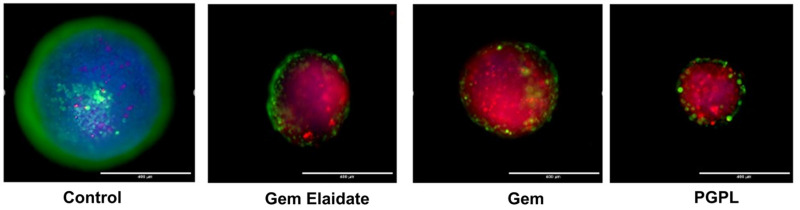
Fluorescence images depicting apoptosis of spheroids treated with control, Gem Elaidate, Gem, and PGPLs. Scale bar: 400 μm.

Using a Live/Dead Cell Assay Kit, the vitality of the cells within the spheroids was assessed with the help of fluorescence microscopy. Figure 9 demonstrates that, when compared to the control, which exhibited dominant green fluorescence, spheroids treated with Gem Elaidate and PGPLs included a greater percentage of dead cells, as indicated by the red fluorescence. Spheroids treated with PGPLs had an increased red fluorescence, an indication of greater cytotoxicity, than those treated with Gem Elaidate alone.

## 4. Discussion

In current clinical practice, Gem is an FDA-approved chemotherapeutic agent for treating pancreatic, breast, lung, and ovarian cancer alone or in combination therapy. Despite all the advancements in Gem research, it is not therapeutically efficient due to its poor pharmacokinetics profile [33]. Gem is rapidly metabolized when administered systemically; consequently, frequent dosing is required to achieve the therapeutic benefit, leading to adverse effects. Along with that, Gem encounters several sequential obstacles, hindering the use of Gem as a hydrophilic molecule, with low bioavailability, poor cellular uptake, and equilibrative nucleoside transporter-dependent entry [34]. We portrayed the anticancer efficacy of Gem Elaidate in a pancreatic cancer cell line, which is synthesized using lipophilic Gem by covalently linking with the elaidic acid to overcome the obstacles of Gem.

Nano-liposomal formulations enable the incorporation of chemotherapeutic drugs with distinct chemical characteristics, increasing anti-tumoral potency, while limiting adverse effects on normal tissues. Conventional liposomes have poor stability and are cleared rapidly by the spleen and liver. Furthermore, transforming liposomes to stealth liposomes (PEGylated Liposomes) using DSPE-PEG2000 not only avoids the reticuloendothelial system (RES) uptake but also prolongs circulation time, improving the stability of the formulated liposomes [35]. For instance, PEGylation of the doxorubicin liposome improved its half-life from minutes to hours. As per Han et al., the encapsulation of paclitaxel in long-circulating liposomes prolongs its biological half-life, leading to improved therapeutic efficacy in vivo [36,37]. In addition, in this manuscript, we investigated Palmitoyl-DL-carnitine chloride (PC) as a Protein kinase C (PKC) inhibitor, which also provides a cationic surface charge to nanoliposomes that increases the cellular uptake and enhances the anticancer potency.

PGPLs were successfully formulated using the modified hydration method to yield a particle size of 81.78 ± 2.31 nm and a PDI less than 0.2. Alavi et al. mentioned that, with the small size of liposomes (400 nm), passive targeting can be achieved by enhanced permeation and retention (EPR), which can help accumulate the drug around the tumor tissue, while minimizing damage to healthy surrounding tissues [38]. Protein kinase C inhibitor–palmitoyl carnitine (PC) is lipophilic in nature, having a lipophilic palmitoyl chain within the lipid bilayer and carnitine head on the outer surface of the liposome, which is responsible for providing a cationic charge, while the tumor vasculatures are negatively charged because of the presence of phosphatidylserine on the surface of the cancerous blood vessels; therefore, cationic liposomes (PGPLs) would preferentially bind there and facilitate its permeation into cells. The zeta potential of PGPLs confirmed the cationic charge imparted by PC. A similar liposomal batch was prepared in the absence of PC. It was found that the particle size of PGPLs without PC is more than 130 nm and the PDI was more than 0.30, while the zeta potential was measured as −46.4 ± 12.8. A previous report posits that Protein kinase C inhibitor–palmitoyl carnitine (PC) in the lipid bilayer results in the reduction in particle size while improving the poly dispersity index of nanocarriers, which is reflected in our results. The stability study of PGPLs showed no significant change in the size, PDI and zeta potential of the prepared liposomes for two months at 4 °C storage.

The results of the in vitro cytotoxicity study of PGPLs in two human pancreatic cancer cells (MIA PaCa-2 and BxPC-3) were promising. In comparison to Gem and PC, PGPLs and Gem Elaidate had lower IC50 values in the cytotoxicity assay, indicating that Gem Elaidate is the cause of the cytotoxicity in PGPLs. Fu et al. found similar findings when they calculated the IC50 values for PC in Vemurafenib-resistant melanoma cell lines (A375R and SK-MEL-28 R) as 23 μM and 26 μM [15]. The IC50 of palmitoyl carnitine in the hepatic cancer cell line was reported by Sonja et al. to be 76 μM, indicating that PC alone lacks the potency to be a cytotoxic agent but can be used as one at greater concentrations [39].

Being a hydrophilic molecule, Gem required a transporter identified as human equilibrative nucleoside transporter-1 (hENT1) for the efficient entry into cells. On the other hand, Gem Elaidate enters independently into pancreatic cancer cells. Our quantitative cellular uptake study is in agreement to demonstrate the lower uptake of Gem compared to Gem Elaidate alone and PGPLs. In contrast, Gem Elaidate alone shows a higher uptake compared to PGPLs, which could be aided by the presence of DMSO. Thus, during 2D cell culture assays, it is possible that the drug–DMSO solution showed a higher uptake than liposomes. During in vivo studies, nanoliposomes show a much higher tumor uptake due to the biodistribution and EPR effect [40]. Our present studies give an idea regarding the quantitative cellular uptake of Gem Elaidate and PGPLs in tumor cells compared Gem.

The Food and Drug Administration (FDA) suggests performing an in vitro hemolysis test for the formulations that need to be administered intravenously. As per Mourtas et al., the study results indicate that conventional lipids such as DOPC, cholesterol, and DSPE-PEG-2000 are safe to use in humans after the intravenous administration of the liposomal formulation. The standardized test method also specifies that, for the product to be safe, the percent hemolysis should not be more than 5% [41,42]. Indeed, our results depict that PGPLs, when analyzed at a Gem Elaidate concentration range of 5–20 µM, show minimum disruption of RBCs (<1%) in comparison with the positive control (SDS) and are dispersible within the plasma. Hence, it is safe for IV administration.

The Extracellular Matrix (ECM) proteins of pancreatic cancer play a crucial role in tumorigenesis. The effect of a different treatment group on cancer cell migration and metastasis was evaluated using a two-dimensional (2D) surface migration assay [43]. The in vitro bridging of MIA PaCa-2 cells in the presence of Gem Elaidate (68% bridging of migration area) was observed at 100 nm, which explains its role in the inhibition of metastasis for the treatment of pancreatic cancer. Moreover, the PGPL treatment group resulted in a 46% reduction in the percentage bridging migration potential of cells compared to control cells, while only an 80% reduction in migration capability was seen when treated with Gem alone. Furthermore, the surrounding cells in the wells were not affected, with the treatment group further verifying that the reduction in the bridging area is not due to the cytotoxic effects of the drug but it is due to the inhibition of cell migration. Further, the clonogenic assay was performed to determine whether there are metastatic-resistant pancreatic progenitors to quantify their ability to proliferate and differentiate into colonies in a six-well plate. Firstly, the capacity of pancreatic cells to develop colonies was determined, and then the effect of Gem Elaidate, Gem, and PGPL treatments on the clonogenicity of MIA PaCa-2 cells was investigated. The results demonstrate that Gem Elaidate counteracts the growth and metastasis of the aggressive MIA PaCa-2 cells at a non-cytotoxic concentration (50 nM). Moreover, PGPLs significantly inhibited the formation of a number of colonies and led to a reduction in the colony area compared to Gem Elaidate. Furthermore, the percent colony formation efficiency of PGPLs reduced 3.2-fold compared to Gem Elaidate and 4.9-fold compared to Gem-treated cells. Hence, the results obtained from in vitro cytotoxicity, migration, and clonogenic assays illustrate that Gem Elaidate effectively inhibits the proliferation, migration, and metastasis of MIA PaCa-2 pancreatic cancer cells, and formulated nanoliposomes (PGPLs) further enhanced its anticancer capability. The expression of PKCα, PKCβ1, and PKCδ is elevated in PDAC, whereas that of PKCε is elevated in normal tissue. Moreover, PKCα is believed to be one of the biomarkers for the diagnosis of pancreatic cancers. On the overexpression of PKCα, the survival rate is decreased because it is directly related to the cell proliferation of pancreatic cancer cells. It was reported that PKC is essential for vasculogenic tube formation, so the inhibition of PKC can help in pancreatic cancer cell invasion [44,45]. Anti-pancreatic cancer vasculogenic mimicry agents are inadequate, so Palmitoyl-DL-carnitine chloride (PC) was explored as a Protein kinase C (PKC) inhibitor in the MIA PaCa-2 pancreatic cancer cell line. Using PC, the inhibition of vasculogenic mimicry channel formation was previously seen in melanoma cancer cell lines (A375 and A375R) using a PKC inhibitor [15,46]. Similar results were obtained for the PGPL treatment group in MIA PaCa-2 pancreatic cancer cells. From our western blot results, we found that Gem Elaidate significantly reduced the levels of anti-apoptotic protein Bcl-2, while increasing the level of apoptotic marker-cleaved caspase-3 and P53. Our findings are consistent with those of Patki et al., who claimed that Gem induces apoptosis in MIA PaCa-2 pancreatic cell lines regulating members of the Bcl-2, which is an anti-apoptotic protein [27]. According to our western blot data, the expression of cleaved caspase-3 was also found to be higher in cells treated with PGPLs than in those treated only with Gem Elaidate.

The development of 3D culture models has proven to be a valuable and versatile tool for anticancer drug screening, but also for gaining mechanistic insight into the regulation of cancer cell death and viability under conditions imitating those in the tumor microenvironment. Cell proliferation has a pivotal effect for anticancer drugs, which seems to reduce in 3D compared to 2D assay conditions; therefore, it is important to check the activity in 3D tumor spheroids rather than 2D cell culture. In the Gem Elaidate treatment group, the spheroid area reduced, indicative of cell death, until the fourth day. The growth as sustained until the tenth day of treatment compared to the control group, which showed continual growth. On the contrary, Gem, a polar molecule, has restricted uptake into the cancer cell spheroids. This, therefore, is a reason to explore a lipophilic form of Gem and entrapping Gem Elaidate in nanoliposomes that would most likely improve the anticancer activity of the Gem by protecting it against rapid metabolism and improving its cellular uptake. Furthermore, PGPL treatment reduced the spheroid area in a sustainable manner until day 10 of treatment. Using a Live/Dead Cell Assay Kit and fluorescence microscopy, the cell viability within 3D multicellular tumor spheroids was further examined. Based on the intense red fluorescence of the ethidium homodimer-1 stain compared to the green fluorescence of the calcein acetoxymethyl (AM) stain produced by the control group spheroids, our findings reveal that Gem Elaidate and PGPLs significantly increased apoptosis in the spheroids.

## 5. Conclusions

In conclusion, Gem Elaidate-loaded PEGylated nanoliposomes (PGPLs) were developed by the modified hydration method with significantly higher drug loading, high EE, and a positive surface charge for enhanced anti-pancreatic cancer activity. PGPLs significantly increased pancreatic cancer cell apoptosis and cytotoxicity in 2D and 3D cell culture models. Hence, PGPLs could be an effective and novel nano-formulation for the treatment of PDAC.

## Figures and Tables

**Table 1:** Physicochemical characterization of PGPLs indicating their stability for two months.

Time	Particle Size (nm)	Polydispersity Index	Zeta Potential (mV)
0 day	81.78 ± 12.34	0.150 ± 0.012	+31.6 ± 1.21
7 days	80.12 ± 10.98	0.154 ± 0.034	+31.9 ± 0.98
14 days	83.96 ± 10.12	0.154 ± 0.021	+32.1 ± 2.02
21 days	83.12 ± 14.56	0.159 ± 0.067	+32.7 ± 1.34
1 month	87.01 ± 10.09	0.163 ± 0.014	+32.6 ± 2.17
2 months	88.12 ± 9.10	0.169± 0.019	+31.8 ± 1.97

**Table 2:** IC50 of Gem Elaidate, Gem, and PGPLs in MIA PaCa-2 and BxPC3 pancreatic cancer cell lines.

IC50 (µM)	MIA PaCa-2	BxPC-3
Gem	0.550 ± 0.101	0.614 ± 0.07
Gem Elaidate	0.164 ± 0.08	0.193 ± 0.06
PGPL	0.152 ± 0.05	0.129 ± 0.08
PC	36.62 ± 3.895	31.79 ± 4.161

## Data Availability

The data presented in this study are available on request from the corresponding author.

## Supplementary Materials

The following supporting information can be downloaded at: https://www.mdpi.com/article/10.3390/cancers15010182/s1, Figure S1: Physicochemical characterization of developed liposomes without PC—(a) Dynamic light scattering graphs illustrating unimodal particle size distribution and (b) Positive ζ-potential of liposomes without PC; Figure S2: In vitro cytotoxicity measured by MTT assay after 48 h treatment with concentrations ranging from 0.04 uM to 5.0 uM of Gem Elaidate, Gem, and PGPL. (a) and (b) represent the data for MiaPaCa-2 and BxPC-3 cells; Figure S3: Quantitative analysis of intracellular amount of Gem Elaidate alone, Gem alone, and Gem Elaidate in PGPL using BxPC-3 cells incubated for 4 h at 37 °C. (*n* = 3) * *p* < 0.1; ** *p* < 0.01; Figure S4: Uncropped Western blots.
