# Correction: Patel et al. Palmitoyl Carnitine-Anchored Nanoliposomes for Neovasculature-Specific Delivery of Gemcitabine Elaidate to Treat Pancreatic Cancer. *Cancers* 2023, *15*, 182

**DOI:** 10.3390/cancers17010044

**Published:** 2024-12-27

**Authors:** Akanksha Patel, Aishwarya Saraswat, Harsh Patel, Zhe-Sheng Chen, Ketan Patel

**Affiliations:** College of Pharmacy and Health Sciences, St. John’s University, Queens, NY 11439, USA; akanksha.patel21@my.stjohns.edu (A.P.); aishwarya.saraswat19@my.stjohns.edu (A.S.); harsh.patel17@my.stjohns.edu (H.P.); chenz@stjohns.edu (Z.-S.C.)

In the original publication [1], an error was identified in Figure 8. Specifically, duplicate images were inadvertently included for the Day 6 gem group and the Day 10 PGPL group. This error occurred during the selection and compilation of images by the authors. The revised, accurate Figure 8 is provided below. The associated text and figure legend remain unchanged. The authors state that the scientific conclusions are unaffected. This correction was approved by the Academic Editor. The original publication has also been updated.

## Figures and Tables

**Figure 8:**
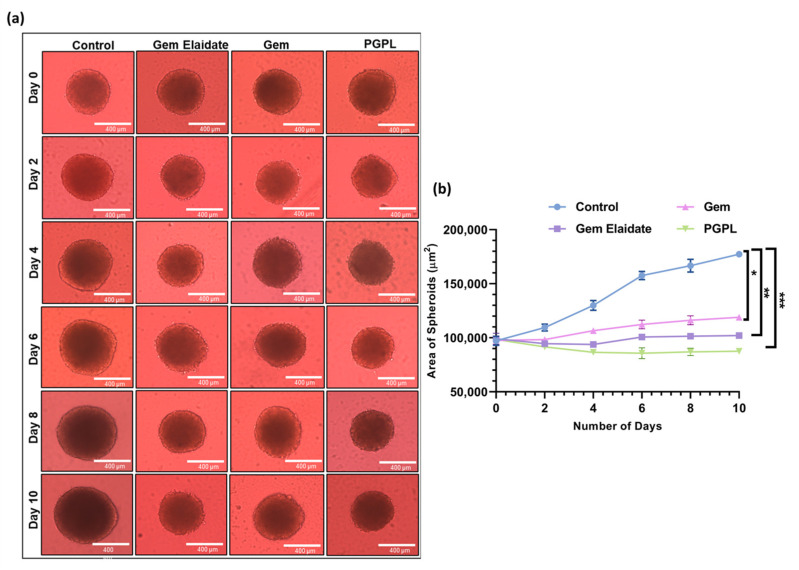
Results for cell viability within 3D multicellular pancreatic tumor spheroids of MIAPa-Ca-2. (**a**) Representative images of spheroids treated with Gem Elaidate, Gem, and PGPLs following 10 days of treatment. (**b**) Area of spheroids as a function of time with various treatment groups. (*n* = 3). * *p* < 0.1; ** *p* < 0.01; *** *p* < 0.001.
