# Supplementary material for: Palmitoyl Carnitine-Anchored Nanoliposomes for Neovasculature-Specific Delivery of Gemcitabine Elaidate to Treat Pancreatic Cancer
Source: Cancers (Basel). 2022 Dec 28;15(1):182. doi: 10.3390/cancers15010182 (PMC9818435; doi:10.3390/cancers15010182)
Supplement: Supplementary file 1 [file cancers-15-00182-s001.zip › cancers-1989360-supplementary.pdf]

cancers

MDPI

Article

Supplementary Materials: Palmitoyl Carnitine-anchored nanoliposomes for neovasculature-specific delivery of Gemcitabine Elaidate to treat Pancreatic cancer

Akanksha Patel, Aishwarya Saraswat, Harsh Patel, Zhe-Sheng Chen and Ketankumar Patel

1. Materials:

3-(4,5-dimethylthiazol-2-yl)-2,5- diphenyl tetrazolium bromide (MTT) was acquired from Fisher Scientific. Crystal violet was purchased from Fisher Scientific (NH, USA). Fetal Bovine Serum (FBS) was procured from Atlanta Biologics (Oakwood, GA, USA), Dulbecco's modified Eagle's medium (DMEM) was purchased from Thermo Fisher Scientific Inc. (Waltham. MA, USA), Roswell Park Memorial Institute (RPMI 1640) (2 mM L-glutamine, 10 mM HEPES, 1 mM sodium pyruvate, 4500 mg/L glucose, and 1500 mg/L sodium bicarbonate) (Manassas, VA, USA), Penicillin-Streptomycin-Amphotericin B (PSA) was procured from MP Biomedicals, LLC (Ohio, USA). High-performance liquid chromatography (HPLC) grade water, acetonitrile, and other analytical grade solvents were procured from Fisher Scientific (Hampton, NH, USA).

2. Methods

2.1. HPLC analysis

Chromatographic separation of Gem Elaidate and Gem was achieved using Waters e2695 separation module with 2998 PDA (Photodiode array) detector and InertSustain C18 column (150 mm × 4.6 mm, 5 μM) (DL sciences, CA, USA). Acetonitrile: Water (80:20) was used as the mobile phase at a flow rate of 1 mL/min and an injection volume of 10 μL for Gem Elaidate and Gem, respectively. Empower 3 software was used for monitoring the output signals. The temperature was maintained at 25˚C, and detection was done at 275 nm.

2.2. Preparation of PGPL

The PGPL were prepared using a modified hydration method. Briefly, Gem Elaidate, DOPC, cholesterol, and DSPE-PEG2000 were dissolved in chloroform. The optimized molar ratio of Gem Elaidate: DOPC: cholesterol: DSPE-PEG2000: PC was 1:10:3.5:0.2:1.2. The chloroform solution was then added dropwise to parenteral-grade mannitol (200 μm) with constant stirring at 45 ˚C and left overnight for evaporation of chloroform. The resultant powder was then dispersed in water containing PC at 55˚C, followed by probe sonication (30% amplitude) for 2 min. Gem liposomes (GL) were fabricated by the same method and with the same composition as PGPL for comparison. Also, Gem Elaidate liposomes were prepared with the same composition without PC.

2.3. In vitro cytotoxicity

In vitro cytotoxicity was evaluated using 3-(4,5-dimethylthiazol-2-yl)-2,5-diphenyl tetrazolium bromide (MTT) assay in two pancreatic cancer cell lines (MIA PaCa-2 and BxPC3) for Gem Elaidate and Gem. Cells were seeded in 96-well plates at a density of 5 x 10^3 cells per well and allowed to settle overnight. Next day, the cells were treated with different concentrations ranging from 0.04 uM to 5.0 uM of Gem Elaidate, Gem, and PGPL. The stock solutions were diluted in a cell culture medium at different concentrations. Cells were incubated at 37 °C for 48h. After 48h, MTT colorimetric assay was performed to

measure cell viability. Briefly, MTT dye was prepared in Phosphate buffer saline (PBS) at a concentration of 5 mg/mL in PBS. Cells were incubated with 10 µL of 5 mg/mL MTT solution in each well for 3 h at 37°C, 5% CO2. After 3 h, the media was removed from wells, and formed MTT- formazan crystals were dissolved by adding dimethyl sulfoxide (DMSO). Plates were vigorously shaken, and the quantity of MTT-formazan was determined by measuring the absorbance using Bio-Tek Instruments, Inc. at 570 nm.

2.4. Quantitative cellular uptake assay

For determining the cellular uptake of Gem Elaidate, 5 x 10^5 MIA PaCa-2 cells and 7.5 x 10^5 BxPC3 cells per well were seeded in a 6-well plate and then treated with Gem Elaidate, Gem, and PGPL (50 µM) for 4 h at 37˚C. Following this, the cells were washed twice with PBS and lysed by adding 0.5% sodium dodecyl sulfate (SDS) to release the intracellular Gem Elaidate and Gem. Samples were then diluted with acetonitrile, centrifuged at 13,300 x g for 10 min, and the supernatant was analyzed by HPLC to investigate the intracellular Gem Elaidate and Gem concentration in the drug and PGPL treated group.

2.5. In vitro hemolysis study

In vitro hemolysis study was carried out using mice's red blood cells (RBCs). C57BL/6 mice (5–6 weeks old) were received from Jackson laboratories (CT, USA). Briefly, mice were anesthetized with 2.5% isoflurane, followed by one-time blood collection using the cardiac puncture technique. Animals were immediately euthanized by carbon dioxide. The experimental protocol was approved by the St. John's University Institutional Animal Care and Use Committee for collection of blood from mice for laboratory use. Cells were separated from plasma by centrifugation at 2000 rpm for 5 min. The cell pellet was then washed twice, followed by redispersion into an appropriate volume of PBS to achieve the same hematocrit. Then, PGPL was dispersed in the RBC aliquots to achieve 5, 10, and 20 µM of Gem Elaidate, respectively. Samples were incubated for 30 min at 37◦C, after which they were centrifuged at 2000 rpm for 5 min. The supernatants were diluted with PBS and analyzed for hemoglobin release using a UV spectrophotometer at 550 nm. PBS was used as the negative control, and sodium dodecyl sulfate solution was used as the positive control (100% hemoglobin release). Percentage hemolysis was calculated by following formula:

$$\%\,\boldsymbol{Hemolysis} = \frac{[\boldsymbol{Absorbance\ of\ test\ sample}] - [\boldsymbol{Absorbance\ of\ negative\ control}]}{[\boldsymbol{Absorbance\ of\ positive\ control} - \boldsymbol{Absorbance\ of\ negative\ control}\,]} * \mathbf{100}$$

2.6. In vitro migration assay

For this assay, MIA PaCa-2 cells were seeded at a density of 10,000 cells per well in a 96-well plate and allowed to attach overnight. After a cells monolayer was formed, a uniform scratch was made in each well using a 200 µL tip. Cells were treated with Gem Elaidate (0.10 µM), Gem (0.10 µM) and PGPL (0.10 µM) for 48 h. Following this, cells were fixed in 4% v/v glutaraldehyde and stained with 0.5% w/v crystal violet. Cells were then washed twice with PBS, and plates were air-dried overnight. Images of the scratch area were captured captured at 10X before and after treatment and analyzed using ImageJ software to determine the percent bridging of migration area for Gem Elaidate, Gem and PGPL treated cells.

2.7. Clonogenic assay

A colony forming assay was performed to determine the effect of Gem Elaidate, Gem, and PGPL on pancreatic cancer cells. Initially, MIA PaCa-2 were seeded at a density of 1 x 10^3 cells per well in a 6-well plate. The cells were treated with Gem Elaidate, Gem and PGPL. Following 24 h of treatment, the cells were allowed to grow until sufficiently large colonies (50 cells/colony) were formed in the control wells. Once the colonies were formed,

cells were fixed using 4% v/v glutaraldehyde followed by staining with 0.5 % w/v crystal violet. Cells were washed twice with HPLC-grade water, and plates were air-dried overnight. The colony counting method was employed for counting the number of colonies and for measuing the area of colony Image J software by NIH was used.

2.8. Western blot assay

Protein expression levels in MIA PaCa-2 cells following treatment were analyzed according to previously established protocol. Briefly, MIA PaCa-2 cells were seeded at a density of 4 × 105 in cell culture flasks of 25 cm2 area. After overnight incubation at 37 °C, cells were exposed to Gem Elaidate and PGPL at a concentration of 200 nM for 48 h, lysates were collected and quantified by total protein concentration using the BCA protein kit (Thermo Scientific, Waltham, MA). An equivalent quantity of total proteins (15 µg) was loaded and separated by SDS-PAGE. Separated bands were transferred onto the PVDF membrane. (Millipore, Billerica, MA). After blocking the membrane in 5% non-fat dry milk (NFDM) in TBST for 90 min at 25 °C, the membrane was incubated with an appropriate concentration of primary antibodies against p53, Bcl-2, cleaved caspase-3, and GAPDH (Cell Signaling Technology, Dancers, MA) at 1:1000 dilution at 4 °C. Later, the membrane was washed with TBST and incubated with horseradish peroxidase-conjugated secondary antibody (Cell Signaling Technology, Dancers, MA) at 1:1000 concentration for 2h at RT. The chemiluminescence signal of the protein-antibody complex was visualized by ECL substrate (Thermo Scientific, Waltham, MA) as per the manufacturer's protocol. GAPDH was used as the loading control and the relative density of each protein band was quantified by ImageJ analysis software (NIH, Bethesda, MD).

2.9. Cell viability within 3D multicellular tumor spheroids

For cell viability and 3D cell imaging on the 11th day, spheroids were stained with a mixture of three dyes: 1 µM calcein AM, 6 µM EthD-1 and 33 mM DAPI (Santa Cruz Biotechnology, Dallas, TX) were prepared in sterile phosphate-buffered saline. Spheroids were incubated with the dye solution for 3 h to allow dye penetration within the spheroids before imaging. Fluorescent images were then captured using an Evos fluorescence microscope (Thermo Fisher Scientific, NH, USA) at 10X magnification.

Results:

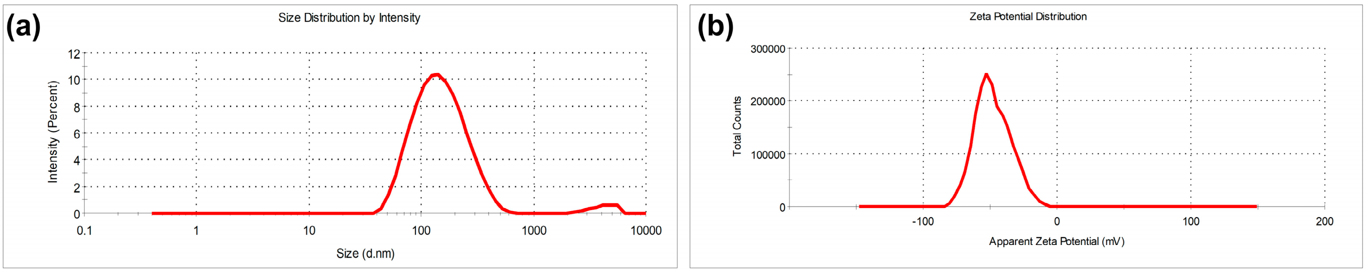

Figure S1. Physicochemical characterization of developed liposomes without PC – (a) Dynamic light scattering graphs illustrating unimodal particle size distribution and (b) Positive ζ-potential of liposomes without PC.

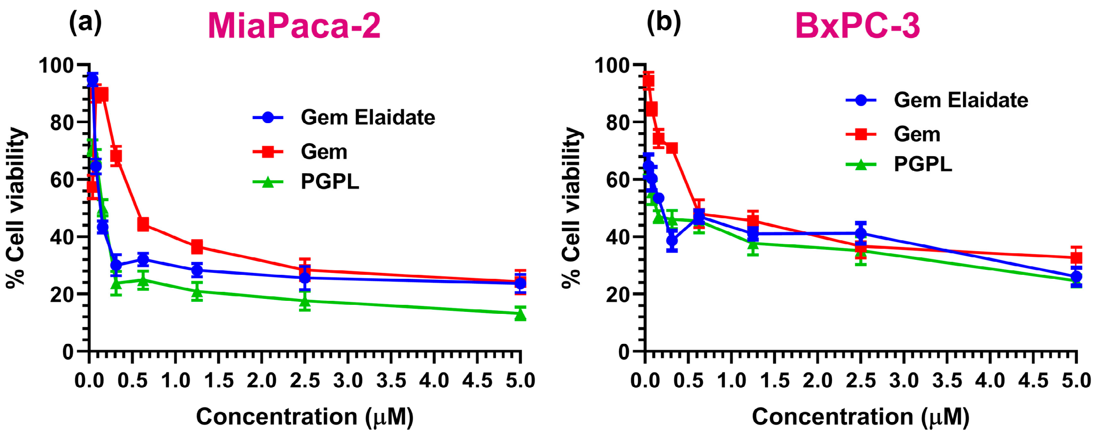

Figure S2. *In vitro* cytotoxicity measured by MTT assay after 48 h treatment with concentrations ranging from 0.04 uM to 5.0 uM of Gem Elaidate, Gem, and PGPL. (a) and (b) represent the data for MiaPaCa-2 and BxPC-3 cells.

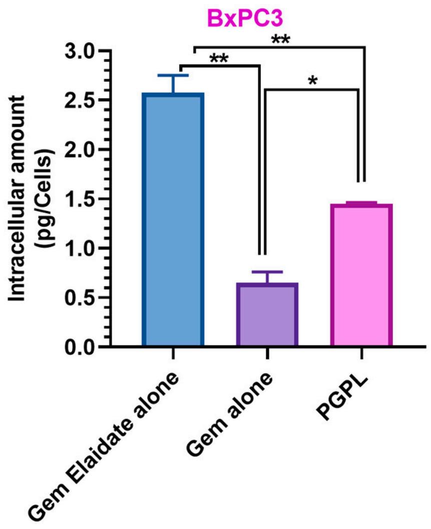

Figure S3. Quantitative analysis of intracellular amount of Gem Elaidate alone, Gem alone, and Gem Elaidate in PGPL using BxPC-3 cells incubated for 4 h at 37°C. (n = 3) *p < 0.1; **p < 0.01.

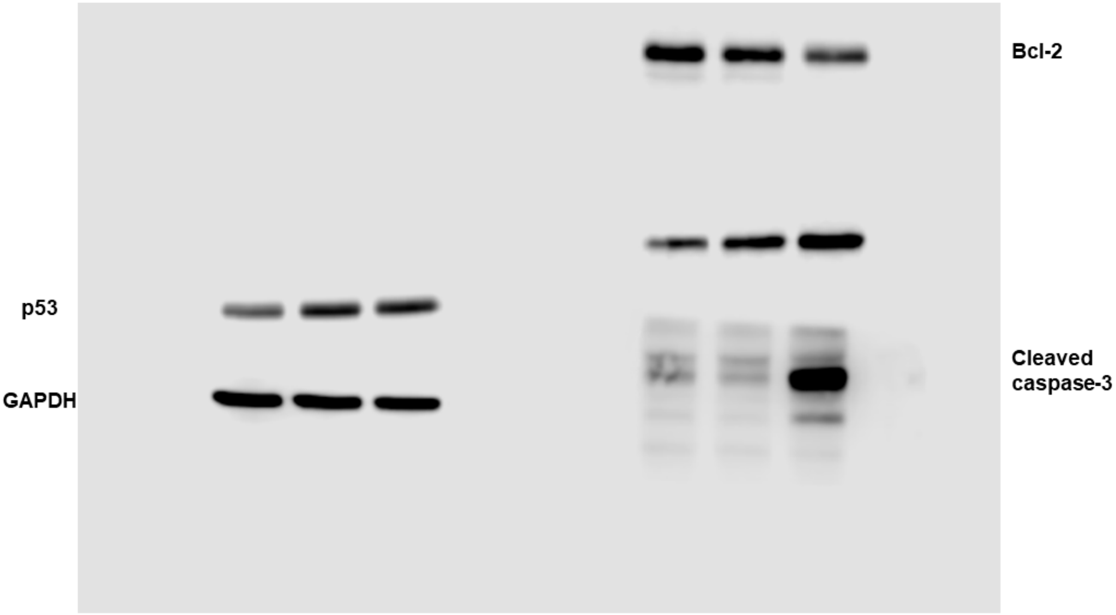

Figure S4. Uncropped Western blots.
